# Significant Enhancement of 5-Hydroxymethylfural Productivity from *D*-Fructose with SG(SiO_2_) in Betaine:Glycerol–Water for Efficient Synthesis of Biobased 5-(Hydroxymethyl)furfurylamine

**DOI:** 10.3390/molecules27185748

**Published:** 2022-09-06

**Authors:** Daozhu Xu, Qi Li, Jiacheng Ni, Yucai He, Cuiluan Ma

**Affiliations:** 1 School of Pharmacy, Changzhou University, Changzhou 213164, China; 2 State Key Laboratory of Biocatalysis and Enzyme Engineering, Hubei University, Wuhan 430062, China

**Keywords:** *D*-fructose, 5-Hydroxymethylfural, 5-(Hydroxymethyl)furfurylamine

## Abstract

5-Hydroxymethyl-2-furfurylamine (5-HMFA) as an important 5-HMF derivative has been widely utilized in the manufacture of diuretics, antihypertensive drugs, preservatives and curing agents. In this work, an efficient chemoenzymatic route was constructed for producing 5-(hydroxymethyl)furfurylamine (5-HMFA) from biobased *D*-fructose in deep eutectic solvent Betaine:Glycerol–water. The introduction of Betaine:Glycerol could greatly promote the dehydration of *D*-fructose to 5-HMF and inhibit the secondary decomposition reactions of 5-HMF, compared with a single aqueous phase. *D*-Fructose (200 mM) could be catalyzed to 5-HMF (183.4 mM) at 91.7% yield by SG(SiO_2_) (3 wt%) after 90 min in Betaine:Glycerol (20 wt%), and at 150 °C. *E. coli* AT exhibited excellent bio-transamination activity to aminate 5-HMF into 5-HMFA at 35 °C and pH 7.5. After 24 h, *D*-fructose-derived 5-HMF (165.4 mM) was converted to 5-HMFA (155.7 mM) in 94.1% yield with *D*-Ala (*D*-Ala-to-5-HMF molar ratio 15:1) in Betaine:Glycerol (20 wt%) without removal of SG(SiO_2_), achieving a productivity of 0.61 g 5-HMFA/(g substrate *D*-fructose). Chemoenzymatic valorization of *D*-fructose with SG(SiO_2_) and *E. coli* AT was established for sustainable production of 5-HMFA, which has potential application.

## 1. Introduction

With the continuous growth in population and the high dependence on non-renewable resources, it is a vital step to switch to alternative clean and renewable energies [1]. Among varied renewable resources, lignocellulosic biomass (LCB) continues to attract much attention as an inexpensive and renewable alternative to fossil fuel to manufacture biofuel molecules and biobased compounds [2]. Biomass has been widely used as feedstock to produce a series of valuable compounds, such as biofuels, biopolymers, energy-rich chemicals, and bioactive molecules [3]. Consequently, there has been a growing interest in the cost-effective valorization of mono- and poly-saccharides into value-added platform compounds in biorefinery processes [4].

5-Hydroxymethylfural (5-HMF), which can be prepared via the dehydration of biomass-derived monosaccharide (e.g., *D*-fructose), is one of the foremost promising platform chemicals [5]. Generally, a number of homogeneous and heterogeneous acid catalysts widely catalyze dehydration of carbohydrates to generate 5-HMF [6]. Recently, the preparation of 5-HMF from carbohydrates has mostly employed corrosive inorganic acids (e.g., HCl, H_2_SO_4_, etc.), preparation of complicated and expensive solid acids, and non-environment friendly metal catalysts [7]. Hence, it is urgent to explore new sustainable catalysts that are in line with the concept of green chemistry to manufacture 5-HMF from carbohydrates [8].

5-HMF has strong reactivity, due to the presence of three functional groups (a furan ring, hydroxy group and an aldehyde group), which can be further valorized into a series of value-added furans [9,10]. 5-(Hydroxymethyl)furfurylamine (5-HMFA), as an important 5-HMF derivative, is utilized for manufacturing drugs, but can also be used as a curing agent for epoxy resins [11]. Typically, 5-HMFA has been mainly synthesized by means of a chemo-catalysis route [5], which usually employs inexpensive, or unfriendly, metal catalysts. In addition, chemo-catalysis can suffer from energetic consumption issues under the harsh performance conditions (e.g., high pressure, high temperature, etc.) [12]. Very recently, bio-catalysis has gained great interest to prepare 5-HMFA, due to the mild reaction conditions, good catalytic activity, excellent selectivity, and eco-friendliness [13].

The strategy for bridging nonenzymatic and enzymatic catalysis has been well established for the production of value-added building-blocks [14,15]. Chemoenzymatic cascade catalysis enables two or more steps in a one-pot manner, which can ignore the separation of intermediates and, thus, reduce performance time and performance cost [16]. In chemoenzymatic conversion, the reaction medium has a vital role in influencing catalytic efficiency. Water is known to be a typical green solvent for preparing 5-HMF and its derivatives [17]. As a polar protic solvent, water can easily cause the occurrence of side-reactions and low productivity of 5-HMF [18]. The past decade has witnessed the expeditious development of deep eutectic solvents (DESs) and their wide applications in chemo-catalysis and bio-catalysis reactions [19,20]. DESs have many advantages, such as extremely low toxicity, good biodegradability, and biocompatibility with enzymes [21,22]. Combining biomass pretreatment and whole cell catalysis for preparation of 5-HMFA in a DES–water medium is a promising strategy for sustainable production of biobased compounds from biomass.

**Figure 1:**
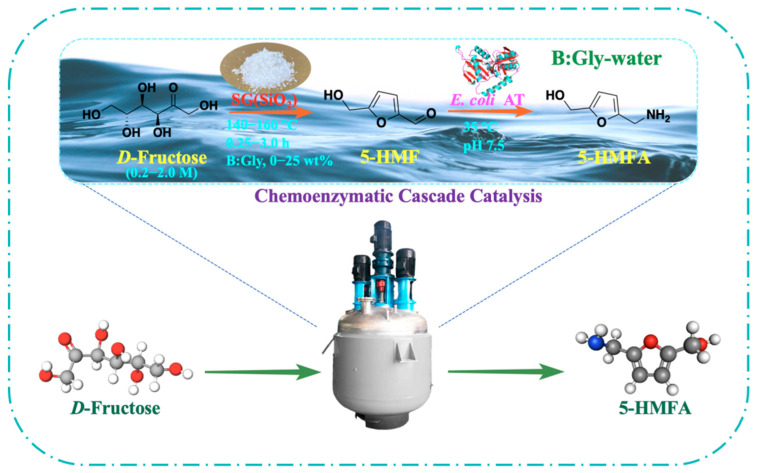
A hybrid synthetic route for the chemoenzymatic sustainable synthesis of 5-HMFA from *D*-fructose in betaine-based DESs–water medium.

Herein, a hybrid synthetic route for producing 5-HMFA from biobased *D*-fructose was constructed in a tandem reaction with SG(SiO_2_) chemo-catalyst and ω-transaminase biocatalyst in a betaine-based DES–water medium (Figure 1). The effect of medium composition, dehydration temperature and duration, catalyst SG(SiO_2_) dose, and *D*-fructose dosage were examined in terms of the dehydration of *D*-fructose into 5-HMF with SG(SiO_2_) chemo-catalyst. Subsequently, recombinant *E. coli* AT containing ω-transaminase [23] was employed to aminate *D*-fructose-derived 5-HMF into HMFA in a DES–water system. Finally, a hybrid reaction with SG(SiO_2_) chemo-catalyst and *E. coli* AT biocatalyst was conducted for sustainable preparation of 5-HMFA from biobased *D*-fructose in eco-friendly DES–water.

## 2. Results and Discussion

### 2.1. Investigation of B:Gly–Water Composition on the 5-HMF Formation

**Figure 2:**
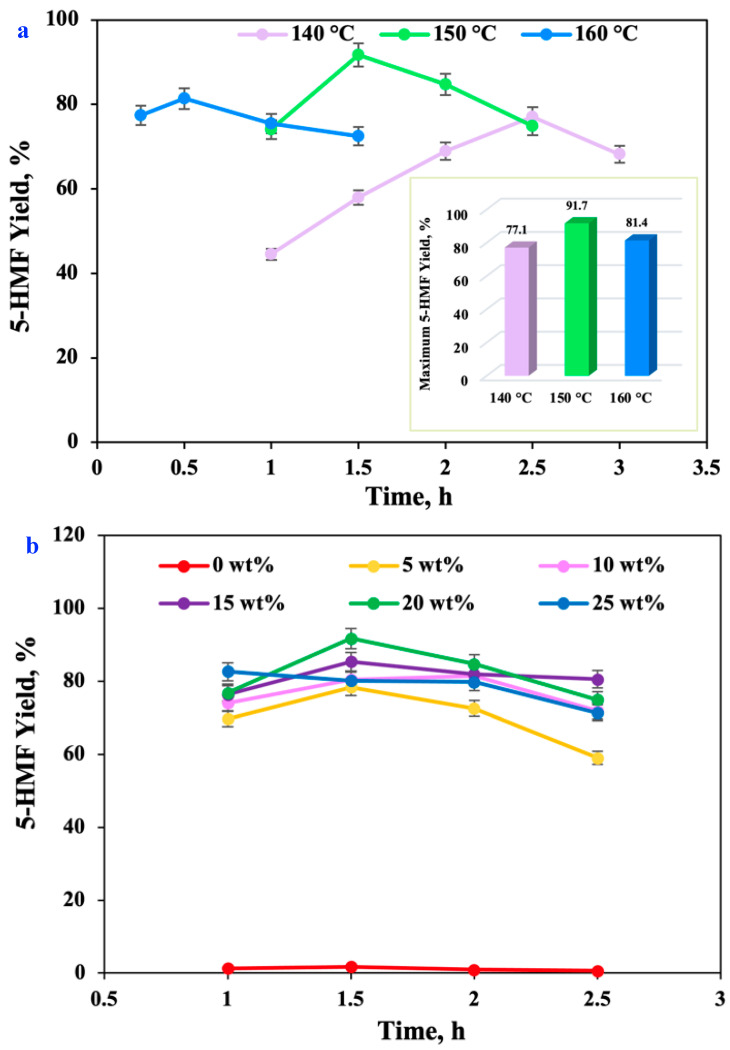
Effect of solvent B:Gly composition (0–25 wt%) on the 5-HMF formation [SG(SiO_2_) 3 wt%, 150 °C, 1–2.5 h] (**a**); Effect of reaction duration (0.25–3 h) and reaction temperature (140–160 °C) on the 5-HMF formation [B:Gly 20 wt%, SG(SiO_2_) 3 wt%] (**b**).

The yield of chemical and biological reactions is largely dependent on the reaction medium [24]. Figure 2a showed the results of SG(SiO_2_) solid acid catalyzing *D*-fructose dehydration to form 5-HMF at 150 °C in various B:Gly–water media. In the aqueous system without DES, the high yield of 5-HMF was only 1.7%. In a certain range of B:Gly–water ratio, the 5-HMF yield was augmented when the B:Gly-to–water mass ratio became higher. After dehydration for 1 h, the 5-HMF yield reached 69.7% [in B:Gly (5 wt%)], 74.0% [in B:Gly (10 wt%)], 76.4% [in B:Gly (15 wt%)], 76.8% [in B:Gly (20 wt%)], and 82.6% [in B:Gly (25 wt%)], implying that the addition of an appropriate amount of B:Gly was favorable for 5-HMF formation. The reason might be that B:Gly might confine the degradation of 5-HMF. During the *D*-fructose dehydration, the yield of 5-HMF showed different trends when the B:Gly–water ratios were different. In the presence of 25 wt% B:Gly, the 5-HMF yield continued to decrease with the increase of dehydration time. At other ratios of B:Gly to water (5:95, 10:90, 15:85, and 20:80; wt:wt), the 5-HMF yield increased first but, then, gradually decreased, reaching the maximum yield of 78.3%, 81.5%, 85.3%, and 91.7%, respectively. *D*-Fructose was dehydrated into 5-HMF in a yield of 78% by Amberlyst 15 chemo-catalyst in ChCl:GA at 60 °C for 4 h [25], and other catalysts and reaction solvents were used to synthesize 5-HMF. WCl6 catalyst converted *D*-fructose into 5-HMF in a 72% yield in THF–[Bmim]Cl medium at 50 °C for 4 h [6]. [C_3_SO_3_Hmim][HSO_4_] could dehydrate *D*-fructose to 5-HMF with a yield of 73% in MIBK–H_2_O, respectively [4]. 5-HMF yield reached 35% by co-catalysis with malic acid and Betaine/HCl at 140 °C for 11 min in H_2_O–ethyl acetate [7]. NbPO dehydrated *D*-fructose to 5-HMF with a yield of 70% [8]. In this work, the maximum yield of 5-HMF was only 1.7% in the aqueous system containing SG(SiO_2_) (Table S1, in Supplementary Materials). In the B:Gly–water system without SG(SiO_2_), the highest yield of 5-HMF was 48.7%. In B:Gly–water (B:Gly, 20 wt%) containing SG(SiO_2_) solid acid catalyst, the 5-HMF yield reached a maximum of 91.7% at 150 °C for 1.5 h. The main reason was that the medium containing B:Gly affected the hydrogen-bond network, and co-catalysis with B:Gly and SG(SiO_2_) could efficiently catalyze the 5-HMF formation from *D*-fructose. Another reason was that many side-reactions would happen in single water system, while 5-HMF was more stable in B:Gly–water.

### 2.2. Effects of Dehydration Temperature and Duration on the 5-HMF Formation

The effect of reaction temperature and duration on the generation of 5-HMF is presented in Figure 2b. After dehydration for 1–2.5 h, the conversions were 100%. The yield of 5-HMF was only 44.5% at 140 °C after this dehydration reaction proceeded for 1 h, whereas the 5-HMF yield jumped up to 77.1% after catalyzing *D*-fructose reacted for 2.5 h. When the dehydration temperature was increased further, the yield of 5-HMF clearly rose to over 80% in a short period of dehydration time. The highest 5-HMF yield at increasing temperatures could reach 81.4% (160 °C, 0.5 h) and 91.7% (150 °C, 1.5 h). These results indicated that the reaction temperature had considerable effects on the generation of 5-HMF and that high temperatures could shorten the time required for the 5-HMF yield to reach its maximum [1]. Although the higher dehydration temperature shortened the duration for the *D*-fructose dehydration to 5-HMF, the secondary reaction against 5-HMF also caused a decrease in 5-HMF yield [26]. Consequently, the optimal dehydration condition for *D*-fructose dehydration to 5-HMF with SG(SiO_2_) catalyst was carried out in B:Gly–water after 1.5 h at 150 °C.

### 2.3. Investigation of SG(SiO_2_) Dose on the 5-HMF Formation

**Figure 3:**
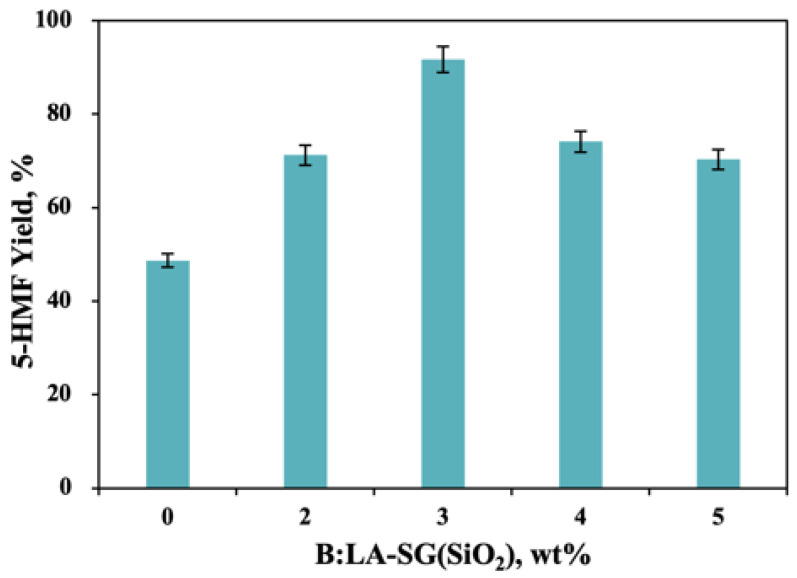
Effect of solid acid dose (SG(SiO_2_) 0–5 wt%) on the 5-HMF formation [B:Gly 20 wt%, 150 °C, 1.5 h].

Figure 3 depicts the effect of catalyst dosage on the 5-HMF yields after the *D*-fructose dehydration reacted at 150 °C for 90 min in B:Gly–water (B:Gly, 20 wt%) system. It had been reported that DESs could convert *D*-fructose to produce 5-HMF as chemo-catalysts and reaction solvents [27]. Without the addition of SG(SiO_2_) to B:Gly–water (B:Gly, 20 wt%) for 90 min at 150 °C, the formed HMF reached a yield of 48.7%. In the presence of a low dose of SG(SiO_2_) catalyst (2 wt%), the productivity of 5-HMF was obviously enhanced, and the yield of 5-HMF reached 71.2%. This might be the role of SG(SiO_2_) solid acid and B:Gly together in catalyzing the generation of 5-HMF from *D*-fructose. As the catalyst SG(SiO_2_) dosage rose from 0 wt% to 3 wt%, the 5-HMF yield obviously increased. Upon raising the dosage of SG(SiO_2_) from 3 wt% to 5 wt%, the 5-HMF yield dropped. The highest 5-HMF yield of 91.7% was achieved using 3 wt% of SG(SiO_2_) as catalyst. *D*-Fructose was transformed to 5-HMF in a dehydration reaction system of DESs (ChCl-phenol)–water with SACS catalyst at 110 °C, achieving a 67% yield of 5-HMF in 4 h [28]. The combination of ChCl:GA (DES) and Amberlyst 15 (catalyst) could dehydrate *D*-fructose to 5-HMF (78% yield) in an aqueous solvent [25]. The above results illustrated that the combination of SG(SiO_2_) solid acid catalyst and B:Gly–water solvent system offered a broad perspective for the economical large-scale production of 5-HMF from *D*-fructose via a dehydration reaction.

### 2.4. Investigation of D-Fructose Dosage on the 5-HMF Formation

**Figure 4:**
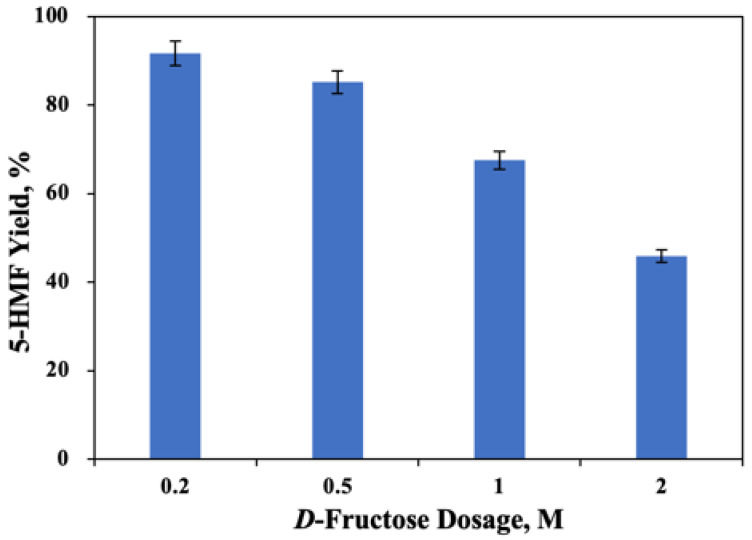
Effect of initial *D*-fructose dose (0.2–2 M) on the 5-HMF formation [B:Gly 20 wt%, SG(SiO_2_) 3 wt%, 150 °C, 1.5 h].

From a practical application point of view, the initial concentration of *D*-fructose is an important aspect to the industrial production of, and economic benefits of, 5-HMF [29]. Hence, different *D*-fructose dosages were applied to the synthesis of 5-HMF (Figure 4). It was clearly ascertained that different initial doses of *D*-fructose had perceptible effects on the 5-HMF yield. When the amount of *D*-fructose increased from 0.2 M to 2 M, the corresponding 5-HMF yield decreased from 91.7% to 45.9%, indicating that *D*-fructose dosage could affect the 5-HMF formation. The reason for this phenomenon might be that the degradation products of *D*-fructose further contributed to the degradation of 5-HMF during the whole reaction [29].

### 2.5. The Recyclability of the Catalytic System

**Figure 5:**
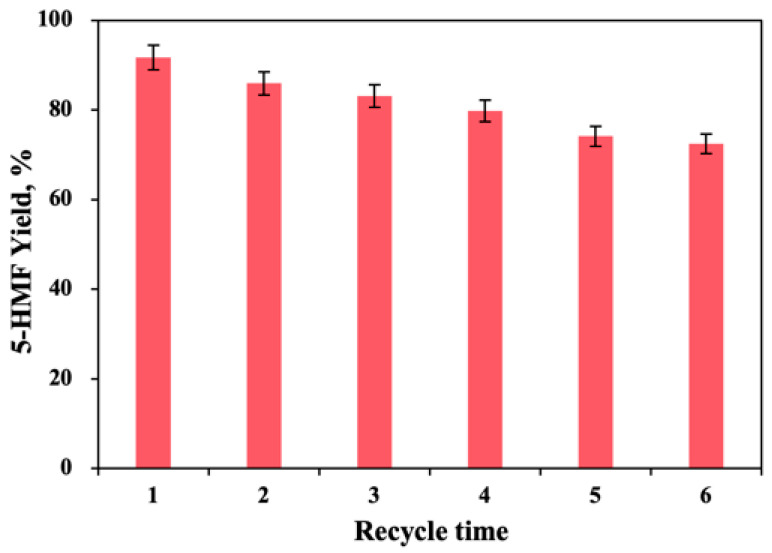
The reuse of the catalytic system on the 5-HMF generation.

The regeneration and reuse of the catalytic system can decrease economic burden and reduce environmental pollution, which is the key to the industrial production of 5-HMF, and has high application potential [30,31]. As presented in Figure 5, the catalytic system was recycled six times in order to test the recyclability of the catalytic system to catalyze the synthesis of 5-HMF from *D*-fructose. 5-HMF was extracted from 5-HMF liquor four times using ethyl acetate by mixing in equal volume. The B:Gly–water medium containing solid acid SG(SiO_2_) was reused for further production of 5-HMF. After the first round, the 5-HMF yield in the catalytic system was 91.7%, and once the three rounds of reaction were over, the 5-HMF yield in the catalytic system decreased slightly to 83.1%. When *D*-fructose was catalyzed from 3rd to 6th batch, 5-HMF yields decreased from 83.1% to 72.4%. The inevitable accumulation of humic substances, could be why the yield of 5-HMF began to decline. In the 6th batch, the catalytic system also efficiently catalyzed *D*-fructose into 5-HMF. When the CrCl_3_-[N_2222_]Cl/EG catalytic system was reused from 1st to 4th batch, the 5-HMF yield dropped from 42% to 38% [32]. These results indicated that the SG(SiO_2_) catalyst with B:Gly–water could be efficiently recycled for *D*-fructose dehydration to produce 5-HMF.

### 2.6. Proposed Catalytic Mechanism for Dehydration of D-Fructose

**Figure 6:**
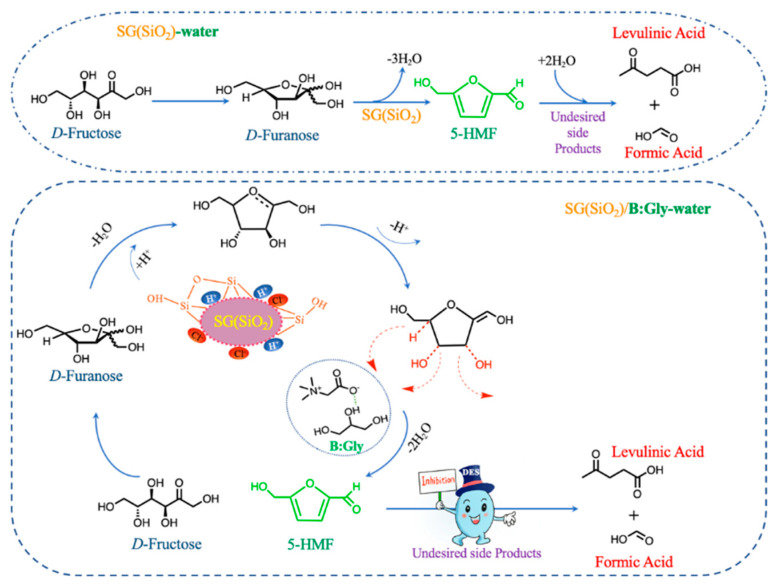
Possible mechanism for the dehydration of *D*-fructose.

A proposed mechanism for SG(SiO_2_)-catalyzed dehydration of *D*-fructose to 5-HMF in a B:Gly–water medium is presented in Figure 6. When fructofuranoses were formed in the reaction medium, the hydroxyl groups on the saturated carbon were rapidly protonated [33]. Due to the presence of H^+^, Cl^−^ and silanol groups, the reaction for removal of the first water molecule would be more effective, which allowed good formation of the enediol intermediates. Upon the action of hydrogen-bonding on C-H and C-O bonds and/or electrostatic forces, the intermediates could facilitate the removal of two water molecules to generate 5-HMF. Electrostatic effects and hydrogen bonding on the polar silanol-rich surface of silica [34], would weaken the stability of C-H bonds and hydroxyl groups in *D*-fructose [35]. This effect might promote the *D*-fructose dehydration by using B:Gly with strong electron-withdrawing ability as the reaction medium. The presence of B:Gly might be beneficial for accepting and donating electrons to improve dehydration of *D*-fructose. Significantly, B:Gly had a great promoting effect on the *D*-fructose dehydration catalyzed with SG(SiO_2_). This effect of hydrogen bonding within the DESs system ultimately improved the *D*-fructose dehydration to 5-HMF.

### 2.7. Biotransformation of 5-HMF into 5-HMFA

**Figure 7:**
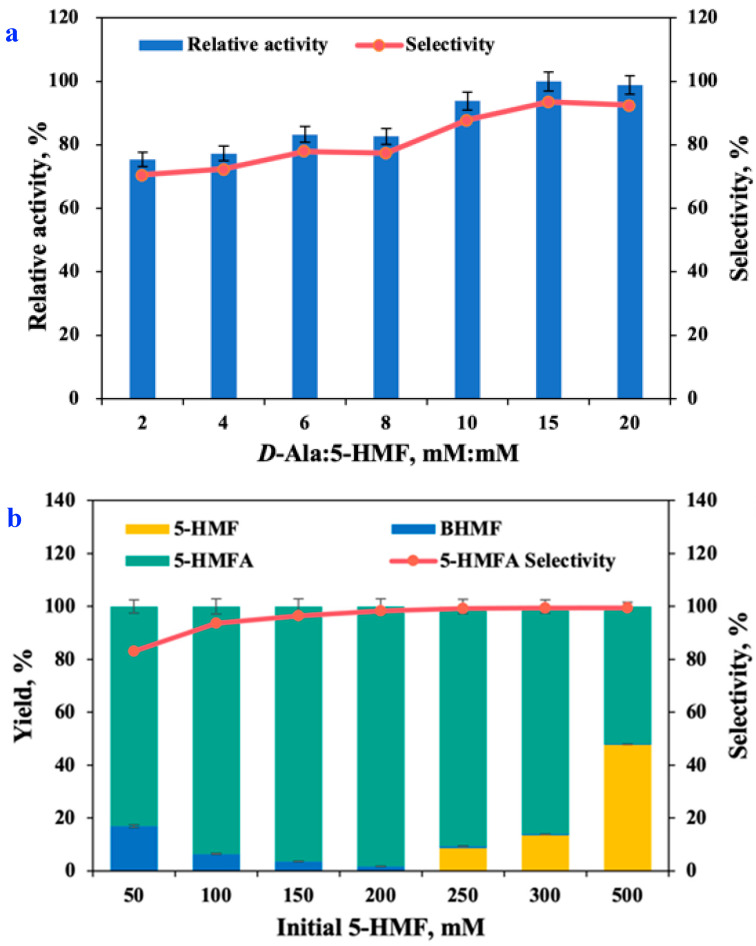
Effects of amine donor *D*-Ala dose (2:1–20:1 of *D*-Ala-to-5-HMF molar ratio) on the yield of 5-HMFA and selectivity [*E. coli* AT 0.050 g/mL, 35 °C, pH 7.5] (**a**); Effects of initial 5-HMF dose (50–500 mM) on the 5-HMFA yield and selectivity [*E. coli* AT 0.050 g/mL, 35 °C, pH 7.5, 15:1 of *D*-Ala-to-5-HMF molar ratio] (**b**).

Bio-catalysis is used to produce high-value chemicals, due to its gentle reaction conditions, few side-reactions and high efficiency of production [36]. Alanine has been considered the most widely used amine donor. In this study, *D*-Ala was used as amine donors during the bio-amination of 5-HMF into 5-HMFA with *E. coli* AT whole cells. Increase of the amine donor dose in the reaction process could be used to alter the reaction balance to generate the product of interest [37]. Figure 7a shows the effect of *D*-Ala dosages on bio-transamination. By increasing the *D*-Ala to 5-HMF molar ratio from 2:1 to 15:1 at 35 °C and pH 7.5 in B:Gly–water (B:Gly, 20 wt%), bio-transamination activity and 5-HMFA selectivity were increased. *D*-Ala and 5-HMF molar ratio of 15:1 was found to provide the optimal reaction conditions. Further increasing the dosages of *D*-Ala in the reaction led to a slight decrease in the bio-catalytic activity and 5-HMFA selectivity, indicating that potential inhibition occurred when the molar ratio of *D*-Ala-to-5-HMF exceeded 15:1 in the bio-transamination reaction.

The tolerance of enzymes to high substrate concentrations is a major process parameter in synthetic biology for the development of efficient biocatalysts [38]. Hence, the substrate tolerance of recombinant *E. coli* AT was investigated under seven different dose of 5-HMF in B:Gly–water (Figure 7b). Interestingly, the selectivity of the product 5-HMFA was low when the substrate 5-HMF dose was low. With the continuous increase of the 5-HMF dose, the selectivity of 5-HMFA also increased. *E. coli* AT could still catalyze the generation of 5-HMFA with high efficiency as the 5-HMF dosage was further increased to 200 mM. By raising the concentration of 5-HMF from 50 mM to 200 mM, the yield of 5-HMFA increased gradually. When the concentration of 5-HMF increased from 200 mM to 500 mM, the yield of 5-HMFA dropped from 98.3% to 52.0%, while the selectivity of bio-amination remained at a high level. Raney Ni was used to chemically catalyze 5-HMF into 5-HMFA (80.7%, yield) under harsh reaction conditions (0.35 MPa NH_3_, 120 °C, and 1 MPa H_2_) [5]. To sum up, *E. coli* AT was able to aminate 5-HMF (200 mM) with *D*-Ala (*D*-Ala-to-5-HMF molar ratio of 15:1) into 5-HMFA with a yield of 98.3% in B:Gly–water (B:Gly 20 wt%, 35 °C, and pH 7.5). Distinct from chemo-catalysis, bio-catalytic synthesis of 5-HMFA from 5-HMF had milder performance conditions, higher yield and easier operation, which was a green and sustainable synthetic route.

### 2.8. Chemoenzymatic Catalysis of D-Fructose to 5-HMFA in B:Gly–Water

**Figure 8:**
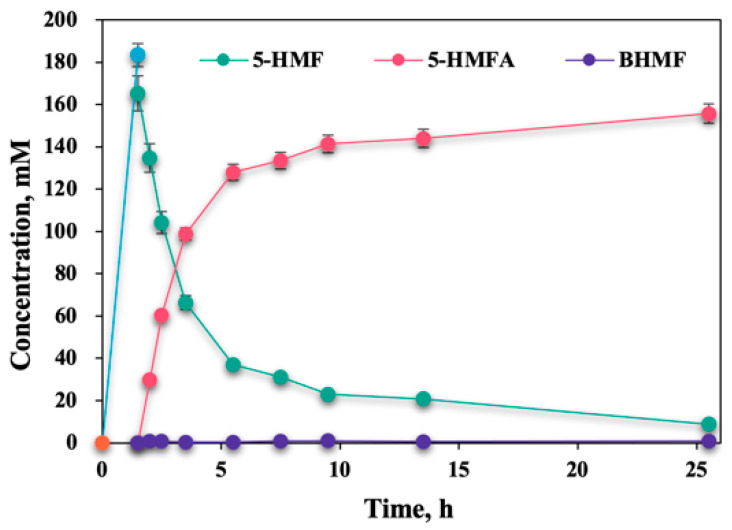
Time courses for chemoenzymatic transformation of *D*-fructose into 5-HMFA.

Chemoenzymatic cascade catalysis, which combines the unique advantages of both non-enzymatic and bio-catalytic reactions, namely the reactivity of chemical catalysts and the high selectivity of enzymes, has become an emerging strategy of interest [38,39]. As illustrated in Figure 8, a combination of chemo-catalysis, using SG(SiO_2_) solid acid, and bio-catalysis, using *E. coli* AT, was used to tandemly convert *D*-fructose into 5-HMFA. An amount of 50 mL B:Gly–water (B:Gly, 20 wt%) containing SG(SiO_2_) (3 wt%) catalyzed 200 mM *D*-fructose at 150 °C to give 183.4 mM 5-HMF after 90 min. The formed 5-HMF liquor was adjusted to pH 7.5 and, then, *E. coli* AT (0.050 g/mL) and *D*-Ala (*D*-Ala-to-5-HMF molar ratio 15:1) were supplemented for the amination of 5-HMF to generate 5-HMFA. Bio-transamination at 35 °C for 24 h, resulted in 165.4 mM of 5-HMF being transformed into 5-HMFA (155.7 mM) in a yield of 94.1%. 5-HMFA ^1^H NMR (CD_3_OD, 400 MHz): δ 1.89 (s, 2H, NH_2_), 3.94 (s, 2H, CH_2_NH_2_), 4.50 (s, 2H, CH_2_OH), 6.27–6.28 (d, *J* = 4.0 Hz, 1H, furan H), 6.32–6.33 (d, *J* = 4.0 Hz, 1H, furan H). Overall, 200 mM *D*-fructose could be converted to 155.7 mM 5-HMFA via a chemoenzymatic approach.

**Figure 9:**
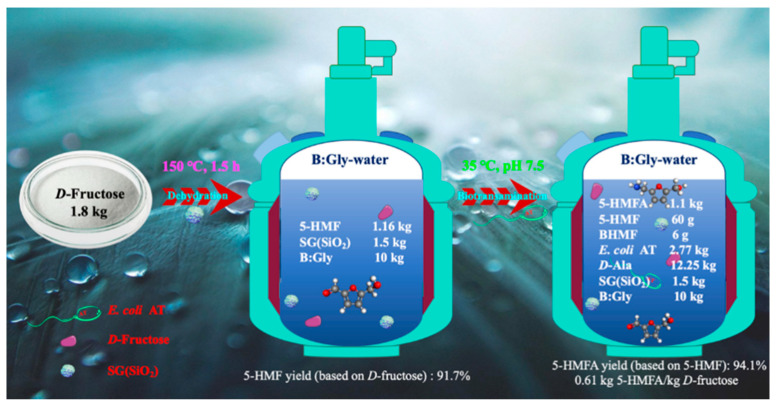
Mass balance from biobased *D*-fructose to 5-HMFA in B:Gly-water.

A mass balance was calculated from *D*-fructose to 5-HMFA in a B:Gly–water system (Figure 9). *D*-fructose (1.8 kg) was dehydrated with SG(SiO_2_) (1.5 kg) and DESs B:Gly (10 kg) under a temperature of 150 °C for 90 min. The resulting 5-HMF liquid containing 1.16 kg of 5-HMF was adjusted to pH 7.5 and then added to *E. coli* AT (2.77 kg) and *D*-Ala (12.25 kg) for biological amination at 35 °C, achieving the productivity of 0.61 kg 5-HMFA/kg substrate *D*-fructose at pH 7.5 in 1 d. Coupling of a biocompatible non-enzymatic catalyst and a highly selective enzymatic catalyst to carry out the dehydration of *D*-fructose into 5-HMFA in B:Gly–water was feasible, which would diminish equipment input, and avoid the separation of intermediates.

**Figure 10:**
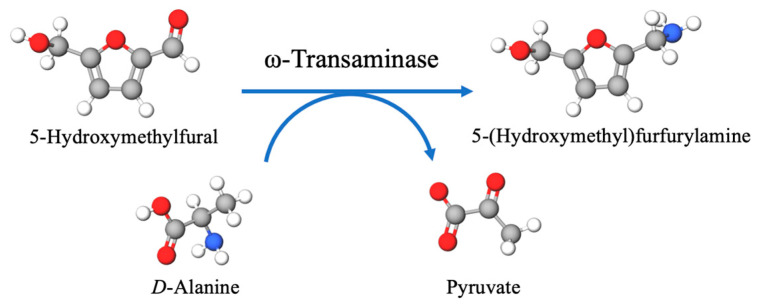
Bioamination of 5-HMF to 5-HMFA.

Chemo-catalysis and bio-catalysis approaches have been used in the synthesis of 5-HMFA [5,40]. 5-HMFA formed in a 4.3% yield from furfurylamine with HCl (6 M) in a formaldehyde solution (35 wt%) at 30 °C after 15 min [40]. Furfurylamine was aminated to 5-HMFA (15% yield) by Amberlyst-15 and formalin at 40 °C after 1 h [41]. Ru/C, Pd/C and Pt/C could catalyze 5-HMF into 5-HMFA with yields of 40.5%, 57.5% and 81.2% under high pressure, respectively [5]. The production of 5-HMFA by the chemo-catalysis technique suffered from several drawbacks, such as unfriendliness, low yield, poor selectivity and harsh performance conditions. In contrast, bio-catalysis has many advantages, such as mild reaction conditions, eco-friendliness and excellent selectivity. The valorization of monosaccharides into 5-HMFA, using chemoenzymatic cascade catalysis, bridges chemical and biological catalysis. In this work, an efficient conversion of biobased *D*-fructose to produce 5-HMF was carried out using SG(SiO_2_) solid acid catalyst in B:Gly–water. During the process of preparing 5-HMF, the reaction medium B:Gly–water played a pivotal role. Relative to the aqueous phase, the addition of DESs could greatly promote the 5-HMF yield. Sequentially, *E. coli* AT cell harboring ω-transaminase was able to aminate *D*-fructose-derived 5-HMF to produce 5-HMFA by using *D*-Ala as amine donor under ambient conditions, accompanied with the formation of pyruvate (Figure 10). To synthesize 5-HMFA, one-pot catalysis of *D*-fructose was carried out by tandem conversion with SG(SiO_2_) and *E. coli* AT cell in B:Gly–water, achieving a remarkable 5-HMFA yield of 86.3% (based on *D*-fructose). Substantially improved productivity was achieved, compared to previous work (5-HMFA yield 64.2%, based on *D*-fructose) [1]. A sustainable strategy for synthesis of 5-HMFA from biobased *D*-fructose was developed in a one-pot manner, which avoided the separation of intermediates and diminished equipment input.

## 3. Materials and Methods

### 3.1. Enzymes, Chemical and Materials

Betaine (B), glycerol (Gly), *D*-fructose, tetraethyl orthosilicate, 5-hydroxymethylfurfural (5-HMF), 5-(hydroxymethyl)furfurylamine (5-HMFA), *D*-alanine (*D*-Ala) and other reagents were obtained from Changzhou Runyou Chemicals Co. (Changzhou, China).

### 3.2. Synthesis of DES B:Gly and Solid Acid SG(SiO_2_)

DES B:Gly was synthesized by heating. Hydrogen-bond-donor glycerol (Gly) and hydrogen-bond-acceptor betaine (B) were well blended in a designed molar ratio (1:2, mol:mol) in an oil bath. After being incubated at 353.15 K by stirring (300 rpm) for 3 h, the formed homogeneous DES solution (betaine:glycerol, B:Gly) was collected. Solid acid catalyst SG(SiO_2_) was prepared as reported previously [20].

### 3.3. Procedure for Conversion of D-Fructose

*D*-Fructose (0.2–2.0 M) and 50 mL B:Gly–water solvent (B:Gly, 0–25 wt%) were blended into a stainless-steel autoclave (100-mL) with SG(SiO_2_) (0–5 wt%) catalysts under agitation (500 rpm) at 140–160 °C after 0.25–3.0 h. Prior to HPLC analysis, the obtained 5-HMF samples were diluted and filtered by means of a 0.22 μm syringe filter.

### 3.4. Recombinant E. coli AT and Its Biotransamination Conditions

Recombinant *E. coli* AT was employed to aminate 5-HMF into 5-HMFA. Cell culture and harvest were carried out as previously reported [23].

To test amine donor on the the influence of 5-HMF bio-transamination, various molar ratio of *D*-Ala-to-5-HMF (2:1–20:1) were loaded in 50 mL reaction medium (100 mM K_2_HPO_4_–KH_2_PO_4_ buffer, pH 7.5) containing B:Gly (20 wt%), 5-HMF (200 mM), and AT cells (0.050 g/mL, wet weight) at 35 °C for 24 h. In order to test the substrate 5-HMF tolerance of AT cells in B:Gly–water medium, 50–500 mM commercial 5-HMF was separately blended with AT cells (0.050 g/mL, wet weight) and *D*-Ala (*D*-Ala-to-5-HMF molar ratio 15:1) in 50 mL reaction medium (100 mM K_2_HPO_4_–KH_2_PO_4_ buffer, pH 7.5) containing B:Gly (20 wt%) at 35 °C for 24 h.

### 3.5. A Hybrid Conversion of D-Fructose from 5-HMFA

Amounts of 200 mM *D*-fructose and 50 mL B:Gly–water solvent (B:Gly, 20 wt%) were blended into a stainless-steel autoclave (100 mL) with SG(SiO_2_) (3 wt%) catalysts by stirring (500 rmp) at 150 °C for 1.5 h. The resulting 5-HMF liquid was adjusted to pH 7.5 and, then, *E. coli* AT (0.050 g/mL, wet weight) and *D*-Ala (*D*-Ala-to-5-HMF molar ratio 15:1) were added to the bio-transamination system to generate 5-HMFA at 35 °C for 24 h.

### 3.6. Analytical Methods

Prior to HPLC analysis, the sample solution was passed through a 0.22-μm milli-pore filter. 5-HMF, 5-HMFA, and BHMF were analyzed on SHIMADZU LC-2030C HPLC system equipped with a Discovery^®^ C18 (4.6 mm × 250 mm, 5 μm) column (Figure S1, in Supplementary Materials). Mixtures containing 20 v% methanol and 80 v% H_2_O containing 0.1% trifluoroacetic acid were utilized as the mobile phase. Column temperature was kept at 35 °C, and the flow rate of the mobile phase was maintained at 0.8 mL/min. 5-HMFA and BHMF were monitored at 210 nm. 5-HMF was monitored at 254 nm.

The 5-HMF yield, 5-HMFA yield, BHMF yield and 5-HMFA selectivity were calculated as follows:

(1) $$\mathrm{Yield}\;\mathrm{of}\;5-\mathrm{HMFA}=\frac{5-\mathrm{HMFA}\;\mathrm{produced}\;(\mathrm{mM})}{\mathrm{Initial}\;5-\mathrm{HMF}\;(\mathrm{mM})}\times 100\%$$

(2) $$\mathrm{Yield}\;\mathrm{of}\;5-\mathrm{HMF}=\frac{5-\mathrm{HMF}\;\mathrm{produced}\;(\mathrm{mM})}{\mathrm{Initial}\;D-\mathrm{fructose}\;(\mathrm{mM})}\times 100\%$$

(3) $$\mathrm{Yield}\;\mathrm{of}\;\mathrm{BHMF}=\frac{\mathrm{BHMF}\;\mathrm{produced}\;(\mathrm{mM})}{\mathrm{Initial}\;5-\mathrm{HMF}\;(\mathrm{mM})}\times 100\%$$

(4) $$\mathrm{Selectivity}\;\mathrm{of}\;5-\mathrm{HMFA}=\frac{5-\mathrm{HMFA}\;\mathrm{produced}\;(\mathrm{mM})}{\left(5-\mathrm{HMFA}+\mathrm{BHMF})\;\mathrm{produced}\;(\mathrm{mM}\right)}\times 100\%$$

## 4. Conclusions

As an important 5-HMF derivative, 5-HMFA is widely used in the production of diuretics, antihypertensive drugs, preservatives and curing agents. In this work, SG(SiO_2_), as a chemo-catalyst, was employed to catalyze *D*-fructose into 5-HMF at 150 °C within 90 min in a B:Gly–water system, achieving the maximum 5-HMF yield (91.7%, based on *D*-fructose). *E. coli* AT catalyzed 5-HMF into 5-HMFA at 35 °C, reaching a remarkable 5-HMFA yield of 94.1% (based on 5-HMF). This was the first example of the efficiently chemoenzymatic synthesis of 5-HMFA from biobased *D*-fructose by a hybrid catalysis with SG(SiO_2_) chemo-catalyst and *E. coli* AT cell bio-catalyst in B:Gly–water, indicating that this strategy has great potential for the manufacturing of 5-HMFA from *D*-fructose under green mild reaction conditions.

## Data Availability

Not applicable.

## Supplementary Materials

The following supporting information can be downloaded at: https://www.mdpi.com/article/10.3390/molecules27185748/s1, Figure S1: HPLC image of 5-HMF (a), BHMF (b), 5-HMFA (c); Table S1: Preparation of 5-HMF from *D*-fructose under different catalytic systems.
