# Supplementary material for: Significant Enhancement of 5-Hydroxymethylfural Productivity from D-Fructose with SG(SiO2) in Betaine:Glycerol–Water for Efficient Synthesis of Biobased 5-(Hydroxymethyl)furfurylamine
Source: Molecules. 2022 Sep 6;27(18):5748. doi: 10.3390/molecules27185748 (PMC9505363; doi:10.3390/molecules27185748)
Supplement: Supplementary file 1 [file molecules-27-05748-s001.zip › molecules-1883579-supplementary.pdf]

Support Information

Figure Caption

Figure S1. HPLC image of 5-HMF(a), BHMF(b), 5-HMFA(c).

Table Caption

Table S1. Preparation of 5-HMF from *D*-fructose under different catalytic systems.

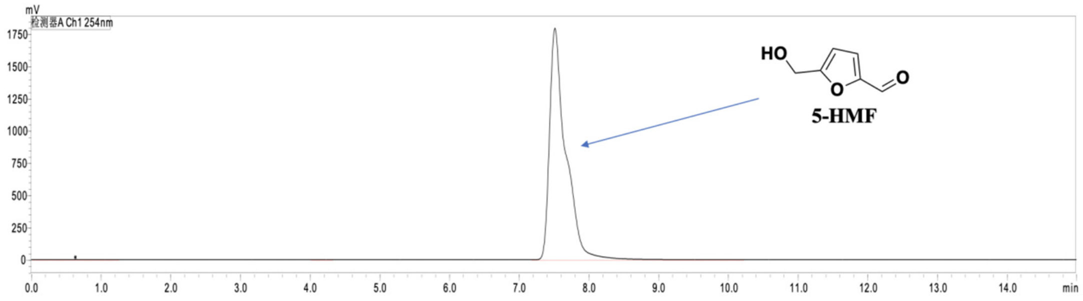

(**a**)

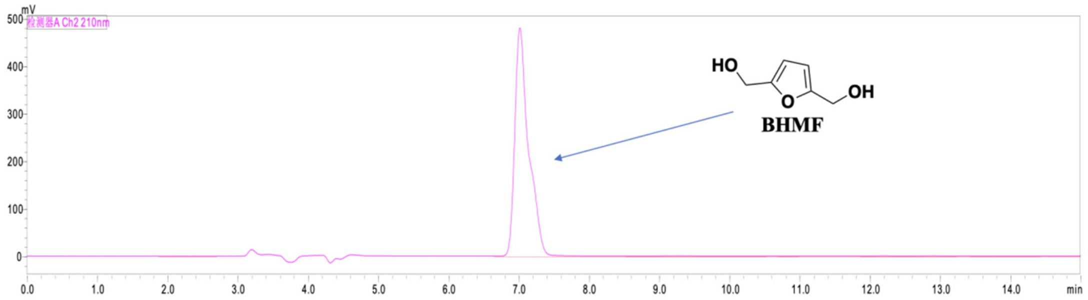

(**b**)

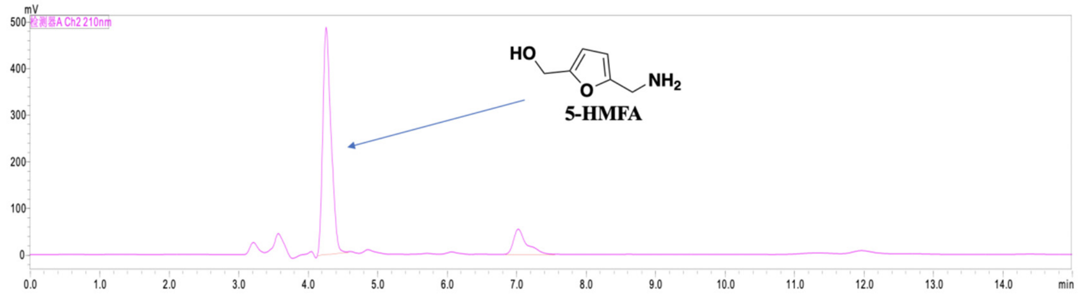

(**c**)

Figure S1. HPLC image of 5-HMF(**a**), BHMF(**b**), 5-HMFA(**c**).

Note: The Chinese in the picture means: Detector.

Table S1. Preparation of 5-HMF from *D*-fructose under different catalytic systems.

Entry	Water	SG(SiO_2)	B:Gly	5-HMF Yield
1	+	+	-	1.7%
2	+	-	+	48.7%
3	+	+	+	91.7%

Condition: B:Gly 20 wt%, SG(SiO_2) 3 wt%, 150 ℃, 1.5 h, reaction system: 50 mL ("+" represents "addition"; "-" represents "no addition").
